# The Mediating Role of Knowledge Sharing in the Relationship Between Transformational Leadership, Organizational Culture, and Nursing Innovation in the Makkah Health Cluster

**DOI:** 10.12688/f1000research.186053.1

**Published:** 2026-07-16

**Authors:** Khadija Lafi Aljarary, Hafizah Che Hassan

**Affiliations:** 1 Faculty of Nursing, Lincoln University College, Petaling Jaya, Selangor, Malaysia

**Keywords:** transformational leadership, organizational culture, knowledge sharing; nursing innovation, registered nurses, healthcare management, PLS-SEM

## Abstract

**Background:**

Nursing innovation is essential for improving healthcare quality, patient safety, and organizational performance. Although transformational leadership and organizational culture have been recognized as important determinants of innovation, the mechanisms through which these organizational factors promote nursing innovation remain insufficiently understood. This study aimed to examine the effects of transformational leadership and organizational culture on nursing innovation, with knowledge sharing as a mediating variable among registered nurses working in the Makkah Health Cluster, Saudi Arabia.

**Methods:**

A quantitative cross-sectional study was conducted among 531 registered nurses selected using stratified random sampling from six hospitals within the Makkah Health Cluster. Data were collected using standardized self-administered questionnaires measuring transformational leadership, organizational culture, knowledge sharing, and nursing innovation. Partial Least Squares Structural Equation Modeling (PLS-SEM) was employed to evaluate the measurement model, structural model, and mediating effects.

**Results:**

Transformational leadership and organizational culture demonstrated significant positive effects on both knowledge sharing and nursing innovation. Knowledge sharing also exerted a significant positive effect on nursing innovation and partially mediated the relationships between transformational leadership and nursing innovation, as well as between organizational culture and nursing innovation. The structural model demonstrated satisfactory explanatory and predictive capability, indicating that leadership, organizational culture, and knowledge-sharing behaviours jointly contributed to nursing innovation.

**Conclusions:**

Transformational leadership, supportive organizational culture, and effective knowledge sharing are complementary organizational resources that promote nursing innovation within integrated healthcare systems. Strengthening leadership capacity, fostering collaborative organizational cultures, and encouraging knowledge-sharing practices may enhance nursing innovation and support ongoing healthcare transformation initiatives under Saudi Vision 2030.

## Introduction

Healthcare systems worldwide are experiencing rapid transformation driven by increasing patient complexity, technological advancement, workforce shortages, and growing expectations for high-quality, patient-centered care. Within this evolving healthcare environment, organizational innovation has become an essential strategy for improving healthcare quality, patient safety, operational efficiency, and organizational sustainability. Innovation in healthcare extends beyond the adoption of new technologies and includes improvements in clinical processes, evidence-based practice, interdisciplinary collaboration, and service delivery models that enhance patient outcomes ( Radaelli et al., 2019; Specchia et al., 2021). As the largest professional group within healthcare organizations, nurses play a pivotal role in generating and implementing innovative practices because they are directly involved in patient care, clinical decision-making, and quality improvement initiatives ( Boamah et al., 2019; Wang et al., 2020). Consequently, strengthening nursing innovation has become a strategic priority for healthcare organizations seeking to achieve excellence in healthcare delivery.

In Saudi Arabia, healthcare transformation under Vision 2030 has accelerated efforts to modernize healthcare services through organizational integration, digitalization, and continuous quality improvement. The establishment of cluster-based healthcare systems, including the Makkah Health Cluster, aims to improve coordination among healthcare institutions while promoting innovation and evidence-based practice across multidisciplinary teams. Achieving these objectives requires healthcare organizations to cultivate leadership approaches and organizational environments that encourage learning, collaboration, and innovation among healthcare professionals.

Among the organizational factors influencing innovation, transformational leadership has received considerable attention because of its ability to inspire, motivate, and empower employees to achieve organizational goals while fostering creativity and professional development. Transformational leaders encourage employees to challenge existing practices, develop innovative solutions, and actively participate in organizational improvement. This leadership style comprises four dimensions, idealized influence, inspirational motivation, intellectual stimulation, and individualized consideration, which collectively create an environment that supports innovation and continuous learning ( Khalili, 2019; Hughes et al., 2018; Bagheri, 2020). Previous studies have consistently demonstrated that transformational leadership enhances employee creativity, organizational commitment, innovation capability, and overall organizational performance, particularly within healthcare settings ( Boamah et al., 2019; Anderson et al., 2020).

Another important organizational determinant of innovation is organizational culture. Organizational culture represents the shared values, beliefs, and behavioural norms that guide employees’ actions and shape organizational practices. A culture characterized by openness, collaboration, trust, and continuous learning encourages employees to exchange ideas, experiment with new approaches, and engage in organizational improvement activities. Innovation-oriented organizational cultures also promote psychological safety, allowing employees to share knowledge and propose creative solutions without fear of criticism or failure ( Hartmann & Herb, 2020; Naranjo-Valencia et al., 2021). Accordingly, organizational culture provides an important foundation for sustaining innovation in complex healthcare environments.

In addition to leadership and organizational culture, knowledge sharing has emerged as a fundamental mechanism through which organizational resources are transformed into innovation. Knowledge sharing refers to the exchange of information, experience, expertise, and clinical knowledge among individuals within an organization, enabling healthcare professionals to learn from one another and collectively solve clinical and organizational problems. Within healthcare organizations, effective knowledge sharing facilitates evidence-based practice, interdisciplinary collaboration, continuous learning, and service improvement ( Nguyen et al., 2021; Islam et al., 2022). Healthcare organizations that promote active knowledge-sharing behaviours are therefore more likely to develop innovative nursing practices and improve healthcare quality.

The proposed relationships among transformational leadership, organizational culture, knowledge sharing, and nursing innovation are supported by several complementary theoretical perspectives. The Knowledge-Based View (KBV) considers organizational knowledge to be a strategic resource that generates competitive advantage and drives innovation through the creation, integration, and application of knowledge. Social Exchange Theory (SET) suggests that supportive leadership and positive organizational environments encourage reciprocal behaviours, including voluntary knowledge sharing among employees. Furthermore, Organizational Learning Theory (OLT) emphasizes that organizational learning processes facilitate continuous knowledge acquisition, knowledge transfer, and innovation. Together, these theoretical perspectives suggest that transformational leadership and organizational culture may foster nursing innovation both directly and indirectly through enhanced knowledge sharing.

Although previous studies have independently demonstrated positive relationships among transformational leadership, organizational culture, knowledge sharing, and innovation, empirical evidence integrating these variables into a single conceptual model remains limited. Existing studies have frequently examined leadership, organizational culture, or knowledge sharing separately, with relatively few investigations evaluating the mediating role of knowledge sharing in explaining nursing innovation. Moreover, evidence from cluster-based healthcare systems in the Middle East, particularly Saudi Arabia, remains scarce despite ongoing healthcare reforms under Vision 2030. Addressing this research gap may provide a more comprehensive understanding of how organizational and social resources jointly contribute to nursing innovation within integrated healthcare systems.

Therefore, this study aimed to examine the effects of transformational leadership and organizational culture on nursing innovation, with knowledge sharing serving as a mediating variable among nurses in the Makkah Health Cluster, Saudi Arabia. By integrating transformational leadership, organizational culture, knowledge sharing, and nursing innovation into a single structural model, this study contributes to the literature on nursing management, organizational behaviour, and healthcare innovation while providing evidence to support leadership development and organizational strategies that foster innovation in healthcare organizations.

## Methods

### Study design

This study employed a quantitative cross-sectional design to examine the relationships among transformational leadership, organizational culture, knowledge sharing, and nursing innovation among registered nurses in the Makkah Health Cluster, Saudi Arabia. A cross-sectional approach was considered appropriate because it enabled the simultaneous assessment of the study variables within a real-world healthcare setting. Partial Least Squares Structural Equation Modeling (PLS-SEM) was used to evaluate both the direct and indirect relationships proposed in the conceptual model. Data analysis was performed using IBM SPSS Statistics version 26 for descriptive analyses and SmartPLS version 4 for measurement and structural model evaluation.

### Study setting

The study was conducted within the Makkah Health Cluster, Saudi Arabia, one of the largest integrated healthcare networks established under Saudi Vision 2030 to improve healthcare quality, coordination, and service integration. The cluster comprises several tertiary and secondary hospitals providing comprehensive healthcare services across multiple clinical specialties. This setting was considered appropriate because it offers a collaborative healthcare environment in which leadership, organizational culture, knowledge sharing, and nursing innovation play important roles in supporting healthcare quality improvement.

### Participants and sampling

The target population comprised all registered nurses employed within the Makkah Health Cluster who were directly involved in clinical care. According to institutional workforce records, the study population consisted of 4,425 registered nurses working in emergency, intensive care, medical-surgical, outpatient, and other clinical departments. Participants were selected using stratified random sampling to ensure proportional representation across participating hospitals and clinical departments.

### Sample size

The minimum required sample size was calculated using Cochran’s formula for finite populations and subsequently adjusted according to the requirements of Partial Least Squares Structural Equation Modeling (PLS-SEM). A final sample of 312 registered nurses was considered adequate to achieve sufficient statistical power while accounting for potential incomplete responses.

### Eligibility criteria

***Inclusion criteria***

Participants were eligible if they: were registered nurses employed within the Makkah Health Cluster; had worked for at least six months in their current organization; and were directly involved in clinical nursing services.

***Exclusion criteria***

Nurses were excluded if they were: undergoing internship or clinical training; on extended leave during the data collection period; or not actively involved in clinical practice.

### Study variables and measurements

Four constructs were assessed using standardized self-administered questionnaires.

***Transformational leadership***

Transformational leadership was measured using a 16-item questionnaire adapted from the Multifactor Leadership Questionnaire (MLQ) developed by Bass and Avolio. The instrument evaluates four dimensions: idealized influence, inspirational motivation, intellectual stimulation, and individualized consideration.

***Organizational culture***

Organizational culture was measured using a 10-item questionnaire assessing organizational values, collaboration, openness, innovation orientation, and adaptability.

***Knowledge sharing***

Knowledge sharing was measured using a 10-item instrument evaluating the exchange of information, experience, professional knowledge, and clinical expertise among nurses in both formal and informal work settings.

***Nursing innovation***

Nursing innovation was assessed using a 10-item questionnaire measuring nurses’ ability to generate, adopt, and implement innovative ideas in clinical practice and healthcare service improvement. All questionnaire items were rated using a five-point Likert scale, ranging from 1 (strongly disagree) to 5 (strongly agree).

### Data collection

Data were collected through an anonymous online questionnaire administered using Google Forms. Before accessing the questionnaire, eligible nurses were provided with an electronic participant information sheet explaining the purpose of the study, study procedures, voluntary participation, confidentiality, and their rights as research participants. Electronic written informed consent was obtained by requiring participants to indicate their agreement before proceeding to the questionnaire. The survey included validated instruments assessing transformational leadership, organizational culture, knowledge sharing, and nursing innovation, together with demographic information such as age, sex, educational level, years of professional experience, and clinical department.

To minimize information bias, all questionnaires were completed anonymously using standardized self-administered instruments. Participants were informed that they could discontinue their participation at any point without consequences. Questionnaires containing incomplete responses were excluded from the analysis, and all study data were securely stored and used solely for research purposes.

### Statistical analysis

Descriptive statistics were calculated to summarize participants’ demographic characteristics and study variables. The measurement model was evaluated by examining indicator reliability, internal consistency reliability (Cronbach’s alpha and composite reliability), convergent validity using the average variance extracted (AVE), and discriminant validity using the heterotrait–monotrait (HTMT) ratio.

The structural model was subsequently evaluated by assessing multicollinearity using variance inflation factors (VIF), path coefficients (β), coefficients of determination (R ^2^), effect sizes (f ^2^), and predictive relevance (Q ^2^). Statistical significance was determined using a bootstrapping procedure with 5,000 resamples. The mediating effect of knowledge sharing was evaluated by examining indirect effects and the variance accounted for (VAF) to determine the extent of mediation.

## Results

### Participant characteristics

A total of 531 registered nurses participated in this study. As presented in Table 1, most respondents were female (71.8%), aged 26–35 years (44.8%), and of Saudi nationality (63.8%). More than half held a bachelor’s degree (52.4%), while staff nurses constituted the largest professional group (70.1%). The Medical/Surgical department represented the highest proportion of respondents (40.3%), and the largest proportion of participants had 4–5 years of clinical experience (25.8%). The respondents were recruited from six hospitals within the Makkah Health Cluster, with the largest proportion coming from Noor Specialist Hospital (27.3%).

**Table 1.** Demographic characteristics of the respondents (N = 531).

Characteristic	Category	n (%)
Age	26–35 years	238 (44.8)
	36–45 years	191 (36.0)
	≥46 years	102 (19.2)
Sex	Male	150 (28.2)
	Female	381 (71.8)
Nationality	Saudi	339 (63.8)
	Non-Saudi	192 (36.2)
Education level	Diploma	139 (26.2)
	Bachelor’s degree	278 (52.4)
	Master’s degree	108 (20.3)
	Doctoral degree	6 (1.1)
Professional position	Staff nurse	372 (70.1)
	Charge nurse	62 (11.7)
	Nurse manager	48 (9.0)
	Nurse educator	29 (5.5)
	Other	20 (3.8)
Department	Emergency	78 (14.7)
	Intensive Care Unit (ICU)	101 (19.0)
	Medical/Surgical	214 (40.3)
	Outpatient	79 (14.9)
	Other	59 (11.1)
Years of experience	1 year	55 (10.4)
	2–3 years	128 (24.1)
	4–5 years	137 (25.8)
	6–7 years	114 (21.5)
	≥8 years	97 (18.3)
Hospital	King Abdullah Medical City (KAMC)	108 (20.3)
	Noor Specialist Hospital (NSH)	145 (27.3)
	Maternity and Child Hospital (MCH)	97 (18.3)
	King Abdulaziz Hospital (KAH)	63 (11.9)
	Hiraa General Hospital (HGH)	60 (11.3)
	King Faisal Hospital (KFH)	58 (10.9)

### Descriptive statistics

The descriptive analysis indicated generally positive perceptions across all study constructs. Transformational leadership demonstrated the highest overall mean score (M = 60.61, SD = 10.22), followed by knowledge sharing (M = 37.83, SD = 6.43), organizational culture (M = 37.79, SD = 6.46), and nursing innovation (M = 37.75, SD = 6.22). Overall, these findings suggest that respondents perceived favorable leadership practices, supportive organizational culture, active knowledge-sharing behaviors, and relatively high levels of nursing innovation within hospitals of the Makkah Health Cluster ( Table 2).

**Table 2.** Descriptive statistics of study variables.

Construct	Mean	SD
Transformational Leadership	60.61	10.22
Organizational Culture	37.79	6.46
Knowledge Sharing	37.83	6.43
Nursing Innovation	37.75	6.22

*Note*: SD = standard deviation.

### Measurement model assessment

The measurement model demonstrated satisfactory psychometric properties. All indicator outer loadings exceeded the recommended threshold of 0.70, ranging from 0.714 to 0.846. Internal consistency reliability was established, with Cronbach’s alpha values ranging from 0.926 to 0.959, while composite reliability values ranged from 0.938 to 0.963. Convergent validity was supported by average variance extracted (AVE) values between 0.602 and 0.643. Furthermore, discriminant validity was confirmed using the Fornell–Larcker criterion and cross-loading analysis, indicating that all constructs were empirically distinct and suitable for structural model evaluation ( Table 3).

**Table 3.** Measurement model assessment.

Construct	Cronbach’s α	Composite Reliability	AVE
Transformational Leadership	0.959	0.963	0.619
Organizational Culture	0.938	0.947	0.643
Knowledge Sharing	0.933	0.943	0.625
Nursing Innovation	0.926	0.938	0.602

Note: Cronbach’s α = Cronbach’s alpha; AVE = average variance extracted.

### Structural model assessment

The structural model demonstrated substantial explanatory and predictive power. The model explained 63.1% of the variance in knowledge sharing (R ^2^ = 0.631) and 62.0% of the variance in nursing innovation (R ^2^ = 0.620). Effect size analysis indicated that transformational leadership exerted a large effect on knowledge sharing (f ^2^ = 0.683), whereas organizational culture demonstrated a moderate effect (f ^2^ = 0.240). The remaining structural relationships showed small-to-moderate effect sizes. Predictive relevance analysis further demonstrated positive Q ^2^ values for both endogenous constructs (knowledge sharing = 0.629; nursing innovation = 0.573), confirming satisfactory predictive capability of the proposed structural model.

### Hypothesis testing

The bootstrapping results demonstrated that all proposed hypotheses were supported ( Table 4). Transformational leadership exerted a significant positive effect on nursing innovation (β = 0.313, *t* = 8.114, *p* < 0.001) and knowledge sharing (β = 0.573, *t* = 20.896, *p* < 0.001). Likewise, organizational culture significantly influenced nursing innovation (β = 0.249, *t* = 7.625, *p* < 0.001) and knowledge sharing (β = 0.340, *t* = 11.048, *p* < 0.001). Furthermore, knowledge sharing demonstrated a significant positive effect on nursing innovation (β = 0.288, *t* = 7.653, *p* < 0.001). Accordingly, all five direct hypotheses (H1–H5) were supported.

**Table 4.** Results of hypothesis testing.

Hypothesis	Structural path	β	t-value	p-value	Decision
H1	Transformational Leadership → Nursing Innovation	0.313	8.114	<0.001	Supported
H2	Transformational Leadership → Knowledge Sharing	0.573	20.896	<0.001	Supported
H3	Organizational Culture → Nursing Innovation	0.249	7.625	<0.001	Supported
H4	Organizational Culture → Knowledge Sharing	0.340	11.048	<0.001	Supported
H5	Knowledge Sharing → Nursing Innovation	0.288	7.653	<0.001	Supported

*Note*: β = standardized path coefficient. Statistical significance was assessed using bootstrapping with 5,000 resamples.

### Mediation analysis

The mediation analysis demonstrated that knowledge sharing significantly mediated the relationships between transformational leadership and nursing innovation as well as between organizational culture and nursing innovation. Because both the direct and indirect effects remained statistically significant and operated in the same direction, the mediation observed in both pathways was classified as complementary (partial) mediation. These findings indicate that transformational leadership and organizational culture enhance nursing innovation not only directly but also indirectly by fostering knowledge-sharing behaviors among nurses. This finding highlights the important role of knowledge sharing as a mechanism through which organizational resources are translated into innovative nursing practices.

## Discussion

This study examined the relationships among transformational leadership, organizational culture, knowledge sharing, and nursing innovation among registered nurses in the Makkah Health Cluster, Saudi Arabia. The findings demonstrated that transformational leadership and organizational culture positively influenced nursing innovation both directly and indirectly through knowledge sharing. These results support the proposed conceptual framework and suggest that leadership, organizational culture, and knowledge-sharing behaviours function as complementary organizational resources that promote innovation within healthcare organizations. Collectively, the findings highlight the importance of integrating leadership, organizational support, and knowledge management to strengthen nursing innovation in healthcare systems undergoing continuous organizational transformation.

One of the principal findings of this study is that transformational leadership significantly enhanced both knowledge sharing and nursing innovation. This finding is consistent with previous studies demonstrating that transformational leaders inspire employees to exchange knowledge, challenge conventional practices, and actively participate in organizational improvement initiatives ( Boamah et al., 2019; Hughes et al., 2018; Bagheri, 2020). Leaders who demonstrate idealized influence, inspirational motivation, intellectual stimulation, and individualized consideration create supportive work environments that encourage nurses to communicate openly, collaborate effectively, and develop innovative approaches to patient care. These findings further reinforce previous evidence indicating that transformational leadership promotes creativity, organizational commitment, and innovation capability while strengthening employees’ willingness to contribute to organizational improvement. From the perspective of Social Exchange Theory (SET), supportive leadership encourages reciprocal behaviours, whereby employees respond to positive organizational treatment by voluntarily sharing knowledge and contributing to innovation.

Another important finding of this study is the positive influence of organizational culture on both knowledge sharing and nursing innovation. Organizational cultures characterized by collaboration, openness, trust, and continuous learning create favourable conditions for employees to exchange ideas, solve problems collectively, and adopt innovative practices. These findings are consistent with previous studies reporting that innovation-oriented organizational cultures facilitate organizational learning, interdisciplinary collaboration, and employees’ readiness to embrace change ( Hartmann & Herb, 2020; Naranjo-Valencia et al., 2021; Shujahat et al., 2021). Within integrated healthcare systems such as the Makkah Health Cluster, a supportive organizational culture may strengthen communication across professional groups and healthcare facilities, thereby creating an organizational environment that continuously supports innovation and quality improvement.

Knowledge sharing emerged as an important mediating mechanism linking transformational leadership and organizational culture with nursing innovation. The findings indicate that leadership and organizational culture not only exert direct effects on innovation but also enhance nurses’ willingness to exchange knowledge, professional experience, and clinical expertise, which subsequently contributes to innovative nursing practices. These findings are consistent with previous studies demonstrating that knowledge sharing enables healthcare professionals to integrate diverse perspectives, improve clinical problem-solving, and facilitate the implementation of innovative healthcare practices ( Nguyen et al., 2021; Islam et al., 2022). The mediating role of knowledge sharing strongly supports the Knowledge-Based View, which considers organizational knowledge to be a strategic resource for creating innovation and sustaining organizational performance. Furthermore, the findings are consistent with Organizational Learning Theory, which emphasizes that continuous learning, knowledge acquisition, and knowledge exchange are fundamental processes through which organizations develop innovative capabilities. Collectively, these findings highlight knowledge sharing as a critical mechanism through which organizational resources are translated into nursing innovation.

This study contributes to the growing literature on nursing management and healthcare innovation by integrating transformational leadership, organizational culture, knowledge sharing, and nursing innovation within a single conceptual framework. Although previous studies have frequently examined these variables independently, the present study provides a more comprehensive understanding of how leadership and organizational culture interact through knowledge-sharing processes to enhance nursing innovation. By integrating the Knowledge-Based View, Social Exchange Theory, and Organizational Learning Theory, this study extends current theoretical understanding of organizational innovation within healthcare settings and provides empirical evidence supporting integrated organizational strategies for strengthening nursing innovation.

### Strengths and limitations

This study has several strengths. First, it simultaneously examined leadership, organizational, and knowledge-management factors using Partial Least Squares Structural Equation Modeling, enabling the evaluation of both direct and indirect relationships among the study variables. Second, validated measurement instruments demonstrated satisfactory reliability and validity, strengthening the credibility of the findings. Third, the study involved a relatively large sample of registered nurses recruited from multiple hospitals within the Makkah Health Cluster, providing comprehensive evidence from an integrated healthcare system undergoing organizational transformation.

Nevertheless, several limitations should be acknowledged. First, the cross-sectional design precludes causal inference among the study variables. Second, the study was conducted within a single healthcare cluster in Saudi Arabia, which may limit the generalizability of the findings to other healthcare settings or countries. Third, all variables were measured using self-administered questionnaires, which may be susceptible to response bias, social desirability bias, and common method variance despite the use of validated instruments.

### Implications for practice

The findings have important implications for nursing managers, hospital administrators, and healthcare policymakers. Healthcare organizations should strengthen transformational leadership through structured leadership development programmes while simultaneously cultivating organizational cultures that promote collaboration, openness, and continuous learning. In addition, hospitals should establish formal and informal knowledge-sharing mechanisms, including interdisciplinary case discussions, mentoring programmes, communities of practice, and digital knowledge-sharing platforms, to facilitate continuous organizational learning and stimulate nursing innovation. Collectively, these organizational strategies may strengthen nurses’ innovative capacity and support the successful implementation of healthcare transformation initiatives under Saudi Vision 2030.

### Future research

Future studies should employ longitudinal or prospective research designs to establish causal relationships among transformational leadership, organizational culture, knowledge sharing, and nursing innovation. Multi-center studies involving different healthcare organizations and healthcare systems are also recommended to improve the generalizability of the findings across diverse healthcare settings. Future research should further investigate additional organizational and psychological factors, including psychological empowerment, innovation climate, organizational commitment, digital leadership, employee resilience, and organizational learning capability, which may provide a more comprehensive explanation of nursing innovation in healthcare organizations. Qualitative or mixed-methods approaches may also provide deeper insights into how nurses experience leadership, organizational culture, and knowledge-sharing practices during organizational change.

## Conclusion

This study demonstrates that transformational leadership and organizational culture are important organizational determinants of nursing innovation within integrated healthcare systems. Both factors exert direct positive effects on nursing innovation while also enhancing knowledge-sharing behaviours, which partially mediate these relationships. These findings highlight the importance of leadership, organizational culture, and knowledge sharing as complementary organizational resources that foster innovation in nursing practice.

From a practical perspective, healthcare organizations should strengthen transformational leadership, cultivate collaborative organizational cultures, and establish effective knowledge-sharing mechanisms to support continuous learning and innovation among nurses. Collectively, these strategies may enhance nursing innovation, improve healthcare service quality, and contribute to the successful implementation of healthcare transformation initiatives under Saudi Vision 2030.

## Ethical considerations

Ethical approval for this study was granted by the Research Ethics Committee (Institutional Review Board) of Lincoln University College, Malaysia (Approval No. LUC/MKT/SP/017/223). Prior to participation, all eligible nurses provided electronic written informed consent. Participation was entirely voluntary, participants could withdraw from the study at any stage without penalty, and strict confidentiality was maintained throughout the research process. No personally identifiable information was collected or retained.

## STROBE checklist

Data are available under the terms of the Creative Commons Attribution 4.0 International (CC BY 4.0) licence.

## Data Availability

The data supporting the findings of this study consist of de-identified questionnaire responses collected from registered nurses working in hospitals within the Makkah Health Cluster, Saudi Arabia. These data have not been deposited in a public repository because they were collected from human participants under conditions of confidentiality and are subject to institutional and ethical restrictions established by the Research Ethics Committee. Under the approved research protocol, participant-level data cannot be released publicly in order to safeguard participant privacy and confidentiality. Researchers interested in accessing the de-identified dataset for legitimate academic or non-commercial research may submit a written request to the corresponding author at khadijalafialjarary@gmail.com. Requests will be evaluated on a case-by-case basis and may require approval from the Research Ethics Committee of Lincoln University College and the participating healthcare institution. Access will be granted only when the proposed use is consistent with the approved ethical protocol and adequate measures are in place to protect participant confidentiality. Zenodo. *Questionnaire for “The Mediating Role of Knowledge Sharing in the Relationship Between Transformational Leadership, Organizational Culture, and Nursing Innovation in the Makkah Health Cluster”* [Data set]. Zenodo. https://doi.org/10.5281/zenodo.21144008 ( Aljarary, K. L., & Hassan, H. C. 2026). This project contains the following underlying data:
• Questionnaire.pdf (Questionnaire containing measurement items for transformational leadership, organizational culture, knowledge sharing, nursing innovation, and demographic characteristics.) Data are available under the terms of the Creative Commons Attribution 4.0 International (CC BY 4.0) licence. This study was reported in accordance with the Strengthening the Reporting of Observational Studies in Epidemiology (STROBE) Statement. Zenodo. The Mediating Role of Knowledge Sharing in the Relationship Between Transformational Leadership, Organizational Culture, and Nursing Innovation in the Makkah Health Cluster: Strobe Checklist [Data set]. Zenodo. https://doi.org/10.5281/zenodo.21099279. ( Khadija Lafi, A., & Hassan, H. C. 2026).
