# The Mediating Role of Knowledge Sharing in the Relationship Between Transformational Leadership, Organizational Culture, and Nursing Innovation in the Makkah Health Cluster

**DOI:** 10.12688/f1000research.186053.2

**Published:** 2026-08-17

**Authors:** Khadija Lafi Aljarary, Hafizah Che Hassan

**Affiliations:** 1 Faculty of Nursing, Lincoln University College, Petaling Jaya, Selangor, Malaysia

**Keywords:** transformational leadership, organizational culture, knowledge sharing; nursing innovation, registered nurses, healthcare management, PLS-SEM

## Abstract

**Background:**

Nursing innovation is essential for improving healthcare quality, patient safety, and organizational performance. Although transformational leadership and organizational culture have been associated with innovation, the mechanisms linking these organizational factors with nursing innovation remain insufficiently understood, particularly within integrated healthcare systems in Saudi Arabia. This study examined the relationships of transformational leadership and organizational culture with nursing innovation and investigated the mediating role of knowledge sharing among registered nurses in the Makkah Health Cluster, Saudi Arabia.

**Methods:**

A quantitative analytical cross-sectional study was conducted among 531 registered nurses recruited using proportionate stratified random sampling from six hospitals within the Makkah Health Cluster. Data were collected using standardized self-administered questionnaires assessing transformational leadership, organizational culture, knowledge sharing, and nursing innovation. Partial Least Squares Structural Equation Modelling (PLS-SEM) was used to evaluate the measurement and structural models and examine direct and indirect relationships among the study constructs.

**Results:**

Transformational leadership was positively associated with nursing innovation (β = 0.313, *p* < 0.001) and knowledge sharing (β = 0.573, *p* < 0.001). Organizational culture was also positively associated with nursing innovation (β = 0.249, *p* < 0.001) and knowledge sharing (β = 0.340, *p* < 0.001). Knowledge sharing was positively associated with nursing innovation (β = 0.345, *p* < 0.001). Knowledge sharing demonstrated complementary partial mediation in the relationships between transformational leadership and nursing innovation (indirect β = 0.198, *p* < 0.001) and between organizational culture and nursing innovation (indirect β = 0.117, *p* < 0.001). The model explained 63.1% of the variance in knowledge sharing and 62.0% of the variance in nursing innovation.

**Conclusions:**

Transformational leadership, organizational culture, and knowledge sharing were significantly associated with nursing innovation, with knowledge sharing representing a complementary pathway linking leadership and organizational culture with innovative nursing behaviour. Healthcare organizations may benefit from integrating leadership development, supportive organizational cultures, and knowledge-sharing strategies to create conditions conducive to nursing innovation and continuous quality improvement within the context of Saudi healthcare transformation.

## Introduction

Healthcare systems worldwide are undergoing rapid transformation driven by increasing patient complexity, rapid technological advancement, workforce shortages, and growing expectations for high-quality, patient-centred care. Within this evolving healthcare environment, organizational innovation has become an essential strategy for improving healthcare quality, patient safety, operational efficiency, and organizational sustainability. Innovation extends beyond the adoption of new technologies and encompasses improvements in clinical processes, evidence-based practice, interdisciplinary collaboration, and service delivery models that enhance patient outcomes ( Radaelli et al., 2019; Specchia et al., 2021). As the largest professional group within healthcare organizations, nurses play a pivotal role in generating and implementing innovative practices because they are directly involved in patient care, clinical decision-making, and quality improvement initiatives ( Boamah et al., 2019; Wang et al., 2020).

In Saudi Arabia, healthcare transformation under Vision 2030 has accelerated organizational reforms aimed at strengthening integrated healthcare delivery through hospital clusters, digital transformation, and continuous quality improvement. The establishment of the Makkah Health Cluster represents one of the major healthcare initiatives designed to improve coordination among healthcare institutions while promoting innovation and evidence-based practice across multidisciplinary teams. Achieving these strategic objectives requires healthcare organizations to cultivate leadership approaches and organizational environments that foster learning, collaboration, and innovation among nursing professionals.

Although previous studies have consistently reported positive associations between transformational leadership, organizational culture, knowledge sharing, and innovation, existing evidence remains fragmented. Most studies have examined these organizational factors independently, while relatively few have investigated how transformational leadership and organizational culture jointly promote nursing innovation through knowledge sharing. Furthermore, empirical findings regarding the mediating role of knowledge sharing remain inconsistent across organizational contexts, and evidence from Saudi Arabia’s cluster-based healthcare system remains limited despite substantial healthcare reforms under Vision 2030. Consequently, a more comprehensive theoretical framework is required to explain how these organizational resources collectively contribute to nursing innovation within integrated healthcare organizations.

Therefore, this study aimed to examine the influence of transformational leadership and organizational culture on nursing innovation, with knowledge sharing serving as a mediating variable among registered nurses working within the Makkah Health Cluster, Saudi Arabia. The theoretical rationale and hypothesis development are presented in the following section.

## Theoretical background and hypothesis development

### Transformational leadership and nursing innovation

Transformational leadership has been widely recognized as one of the most influential leadership approaches for promoting organizational effectiveness and innovation. According to Bass and Avolio, transformational leaders inspire followers to transcend personal interests by articulating a compelling vision, stimulating intellectual curiosity, encouraging innovative thinking, and providing individualized support that enhances professional growth. This leadership style comprises four interrelated dimensions, idealized influence, inspirational motivation, intellectual stimulation, and individualized consideration, which collectively foster an organizational climate conducive to creativity, learning, and continuous improvement ( Bass & Avolio, 1994; Khalili, 2019; Hughes et al., 2018). Within healthcare organizations, transformational leadership is particularly important because nurses routinely face complex clinical challenges that require adaptive thinking, collaborative problem-solving, and evidence-based innovation.

Previous empirical studies have consistently reported that transformational leadership positively influences innovative behaviour among healthcare professionals. Leaders who encourage participation, autonomy, and continuous learning create conditions that motivate nurses to introduce new ideas, improve clinical processes, and actively engage in quality improvement initiatives ( Boamah et al., 2019; Anderson et al., 2020). Similar findings have been reported across hospital settings, where transformational leadership has been associated with increased creativity, organizational commitment, work engagement, and innovation capability among nursing staff. These findings suggest that transformational leadership extends beyond administrative supervision and functions as a strategic organizational resource that enables employees to initiate and sustain innovation.

However, despite the generally positive evidence, previous studies have not produced entirely consistent findings regarding the mechanisms through which transformational leadership promotes innovation. While several investigations have reported direct positive effects of transformational leadership on innovative behaviour, others suggest that leadership primarily exerts its influence indirectly through organizational learning, employee engagement, psychological empowerment, or knowledge-sharing behaviours. These inconsistencies indicate that transformational leadership alone may be insufficient to explain nursing innovation and that additional organizational mechanisms should be considered to better understand how leadership translates into innovative practice within healthcare organizations. Furthermore, empirical evidence examining these relationships within integrated healthcare systems in Saudi Arabia remains limited.

The relationship between transformational leadership and nursing innovation can be explained through both the Knowledge-Based View (KBV) and Social Exchange Theory (SET). The KBV proposes that organizational knowledge represents a strategic resource capable of generating innovation when leaders facilitate knowledge creation, integration, and application across organizational members. Transformational leaders encourage learning, stimulate intellectual exchange, and create opportunities for knowledge utilization that ultimately support innovation. Meanwhile, SET argues that employees tend to reciprocate supportive leadership behaviours by demonstrating positive discretionary behaviours, including creativity, organizational citizenship, and innovation. Consequently, transformational leadership is expected to foster an organizational environment in which nurses are motivated to contribute innovative ideas and participate in continuous organizational improvement.

Based on the theoretical arguments and empirical evidence presented above, the following hypothesis is proposed:

H1: Transformational leadership positively influences nursing innovation among registered nurses.

### Transformational leadership and knowledge sharing

Knowledge sharing is widely recognized as a critical organizational process through which employees exchange knowledge, professional experience, skills, and expertise to enhance individual and organizational performance. Within healthcare organizations, knowledge sharing facilitates interdisciplinary collaboration, supports evidence-based practice, accelerates problem-solving, and enables continuous professional learning. Because nursing practice depends heavily on communication and collective decision-making, effective knowledge sharing is essential for improving clinical performance and promoting innovation ( Nguyen et al., 2021; Islam et al., 2022).

Transformational leadership has been identified as an important antecedent of knowledge-sharing behaviour. Leaders who inspire a shared vision, encourage open communication, provide intellectual stimulation, and demonstrate individualized consideration create organizational environments in which employees feel psychologically safe to exchange knowledge and collaborate with colleagues. Previous studies have consistently reported that transformational leadership strengthens knowledge-sharing behaviours by fostering trust, organizational commitment, and collaborative learning among employees ( Mittal & Dhar, 2015; Nguyen et al., 2021; Islam et al., 2022). Within healthcare organizations, transformational leaders encourage nurses to discuss clinical experiences, disseminate best practices, and collectively develop solutions to complex patient-care challenges, thereby facilitating organizational learning.

Nevertheless, previous empirical findings suggest that the relationship between transformational leadership and knowledge sharing is not always straightforward. While many studies have demonstrated significant positive associations, others have reported that organizational context, communication climate, organizational culture, and employee trust may influence the extent to which transformational leadership promotes knowledge-sharing behaviours. These findings indicate that leadership alone may not be sufficient to stimulate knowledge sharing unless supported by favourable organizational conditions. Moreover, empirical evidence examining this relationship within Saudi Arabia’s cluster-based healthcare system remains limited, highlighting the need for further investigation.

The positive relationship between transformational leadership and knowledge sharing is supported by both the Knowledge-Based View (KBV) and Social Exchange Theory (SET). From the KBV perspective, organizational knowledge represents a strategic asset that generates competitive advantage when effectively created, integrated, and disseminated among organizational members. Transformational leaders facilitate these processes by encouraging learning, collaboration, and the exchange of professional expertise. Meanwhile, SET proposes that employees reciprocate supportive leadership by engaging in voluntary organizational behaviours that benefit both colleagues and the organization, including knowledge sharing. Consequently, transformational leadership is expected to cultivate a collaborative environment that encourages nurses to exchange knowledge and clinical expertise more actively.

Based on the theoretical arguments and empirical evidence presented above, the following hypothesis is proposed:

H2: *Transformational leadership positively influences knowledge sharing among registered nurses.*

### Organizational culture and nursing innovation

Organizational culture refers to the shared values, beliefs, norms, and behavioural expectations that shape how organizational members interact, make decisions, and accomplish their work. In healthcare organizations, organizational culture extends beyond administrative policies and encompasses an environment that promotes collaboration, trust, openness, continuous learning, and employee empowerment. Such a culture encourages nurses to actively participate in quality improvement initiatives, share innovative ideas, and adopt evidence-based practices that enhance patient care and organizational performance ( Hartmann & Herb, 2020; Naranjo-Valencia et al., 2021).

Previous empirical studies have consistently identified organizational culture as an important determinant of organizational innovation. Innovation-oriented cultures provide employees with psychological safety, managerial support, and opportunities for experimentation, enabling them to introduce creative solutions and continuously improve healthcare services. Several studies conducted in healthcare organizations have demonstrated that supportive organizational cultures are positively associated with innovative work behaviour, employee creativity, organizational commitment, and continuous quality improvement ( Naranjo-Valencia et al., 2021; Hartmann & Herb, 2020). Within nursing environments, cultures that encourage openness, interdisciplinary collaboration, and continuous professional learning have also been associated with greater willingness among nurses to implement innovative clinical practices.

Despite this growing body of evidence, previous findings remain incomplete regarding the mechanisms through which organizational culture promotes nursing innovation. While many studies have reported direct positive relationships, others suggest that organizational culture may exert its influence indirectly by strengthening collaborative behaviours, organizational learning, employee engagement, or knowledge-sharing practices. Furthermore, the influence of organizational culture may vary across healthcare systems because organizational values, leadership structures, and professional interactions differ substantially between countries and healthcare organizations. Consequently, empirical evidence from Saudi Arabia’s integrated healthcare clusters remains limited, and the pathways through which organizational culture contributes to nursing innovation require further investigation.

The relationship between organizational culture and nursing innovation is supported by both the Knowledge-Based View (KBV) and Organizational Learning Theory (OLT). According to the KBV, organizational culture facilitates the creation, integration, and utilization of knowledge that ultimately generates innovation and competitive advantage. Meanwhile, OLT proposes that organizational cultures emphasizing continuous learning, collaboration, and reflective practice create favourable conditions for acquiring new knowledge, improving organizational capabilities, and stimulating innovation. Within healthcare organizations, a supportive organizational culture therefore functions as a strategic organizational resource that encourages nurses to develop innovative ideas, implement evidence-based improvements, and contribute to continuous organizational development.

Based on the theoretical arguments and empirical evidence presented above, the following hypothesis is proposed:

H3: *Organizational culture positively influences nursing innovation among registered nurses.*

### Organizational culture and knowledge sharing

Organizational culture plays a fundamental role in shaping employees’ willingness to exchange knowledge and collaborate within organizations. A supportive organizational culture characterized by trust, openness, mutual respect, and continuous learning creates an environment in which employees feel encouraged to communicate, share expertise, and collectively solve organizational problems. In healthcare settings, where patient care relies heavily on interdisciplinary collaboration and timely information exchange, organizational culture becomes an important determinant of effective knowledge-sharing behaviours ( Hartmann & Herb, 2020; Nguyen et al., 2021).

Previous empirical studies have consistently demonstrated that organizational culture positively influences knowledge sharing across various organizational settings. Cultures that emphasize teamwork, open communication, employee participation, and continuous learning facilitate the dissemination of professional knowledge and encourage employees to contribute their expertise for organizational benefit. Within healthcare organizations, supportive organizational cultures have been associated with greater collaboration among healthcare professionals, increased exchange of clinical knowledge, and improved implementation of evidence-based practices ( Islam et al., 2022; Nguyen et al., 2021). These findings suggest that organizational culture provides an important social environment that enables knowledge to circulate effectively among organizational members.

Nevertheless, previous studies also indicate that the influence of organizational culture on knowledge sharing may vary depending on organizational characteristics and contextual factors. Several studies have suggested that organizational culture alone may not automatically stimulate knowledge-sharing behaviour unless employees perceive sufficient trust, leadership support, and psychological safety within their work environment. Furthermore, differences in organizational structures, cultural values, and healthcare systems may influence the extent to which organizational culture promotes knowledge sharing. Despite the increasing interest in this relationship, empirical evidence from Saudi Arabia’s integrated healthcare clusters remains limited, particularly among nursing professionals undergoing organizational transformation.

The positive association between organizational culture and knowledge sharing can be explained through the Knowledge-Based View (KBV) and Organizational Learning Theory (OLT). The KBV emphasizes that organizational knowledge represents a valuable strategic asset whose effectiveness depends on the organization’s ability to facilitate knowledge creation, integration, and dissemination. Meanwhile, OLT proposes that organizational learning occurs within cultures that encourage collaboration, reflection, communication, and continuous knowledge exchange. Accordingly, healthcare organizations that cultivate supportive organizational cultures are expected to strengthen knowledge-sharing behaviours, thereby enhancing organizational learning and improving innovation capability.

Based on the theoretical arguments and empirical evidence presented above, the following hypothesis is proposed:

H4: *Organizational culture positively influences knowledge sharing among registered nurses.*

### Knowledge sharing and nursing innovation

Knowledge sharing is increasingly recognized as one of the most important organizational processes that facilitates innovation by enabling employees to exchange knowledge, professional expertise, clinical experiences, and practical solutions to workplace challenges. Within healthcare organizations, knowledge sharing promotes interdisciplinary collaboration, accelerates problem-solving, facilitates evidence-based decision-making, and supports continuous professional learning. These processes enable nurses to integrate diverse knowledge sources into clinical practice, thereby enhancing their capacity to generate innovative ideas and implement improvements in patient care ( Nguyen et al., 2021; Islam et al., 2022).

Previous empirical studies have consistently demonstrated that knowledge sharing is positively associated with innovation across various organizational contexts. Employees who actively exchange knowledge are more likely to develop creative solutions, adapt to organizational changes, and participate in continuous quality improvement initiatives. Within nursing practice, knowledge sharing facilitates the dissemination of evidence-based practices, strengthens collaborative learning, and promotes the adoption of innovative clinical interventions. Several studies have reported that healthcare organizations characterized by effective knowledge-sharing practices exhibit higher levels of employee creativity, organizational learning, and innovation capability ( Nguyen et al., 2021; Islam et al., 2022; Wang et al., 2020).

Despite the growing evidence supporting this relationship, previous studies have reported variations in the strength of the association between knowledge sharing and innovation. Some investigations suggest that knowledge sharing alone may not be sufficient to generate innovation unless organizations simultaneously provide supportive leadership, adequate organizational resources, and an innovation-oriented culture. Furthermore, most previous studies have examined knowledge sharing within business or manufacturing organizations, whereas empirical evidence from healthcare organizations, particularly within Saudi Arabia’s cluster-based healthcare system, remains comparatively limited. Consequently, further research is needed to clarify the contribution of knowledge sharing to nursing innovation within integrated healthcare settings.

The relationship between knowledge sharing and nursing innovation is primarily supported by the Knowledge-Based View (KBV) and Organizational Learning Theory (OLT). According to the KBV, organizational knowledge represents a valuable strategic asset that creates competitive advantage when knowledge is effectively shared, integrated, and applied to organizational activities. Innovation emerges when existing knowledge is combined with newly acquired knowledge to generate creative solutions and improve organizational performance. Similarly, OLT proposes that continuous organizational learning depends on the effective acquisition, dissemination, and application of knowledge among organizational members. Through active knowledge sharing, nurses continuously develop professional competencies, improve clinical decision-making, and implement innovative healthcare practices. Therefore, knowledge sharing represents a critical organizational mechanism through which organizational resources are transformed into nursing innovation.

Based on the theoretical arguments and empirical evidence presented above, the following hypothesis is proposed:

H5: *Knowledge sharing positively influences nursing innovation among registered nurses.*

Collectively, the proposed hypotheses suggest that transformational leadership and organizational culture function as organizational resources that directly promote nursing innovation while simultaneously enhancing knowledge-sharing behaviours, which in turn facilitate innovation among nurses. This integrated conceptual model provides a comprehensive framework for understanding the organizational mechanisms that support innovation within Saudi Arabia’s cluster-based healthcare system.

## Methods

### Study design

This study employed a quantitative analytical cross-sectional design to examine the relationships among transformational leadership, organizational culture, knowledge sharing, and nursing innovation among registered nurses working in the Makkah Health Cluster, Saudi Arabia. A cross-sectional design was considered appropriate because it enabled the simultaneous assessment of organizational and behavioural variables within a real-world healthcare setting. The study was reported in accordance with the Strengthening the Reporting of Observational Studies in Epidemiology (STROBE) Statement (von Elm et al., 2007). Partial Least Squares Structural Equation Modelling (PLS-SEM) was employed to evaluate the proposed measurement and structural models. Data management and descriptive analyses were performed using IBM SPSS Statistics version 26 (IBM Corp., Armonk, NY, USA), whereas structural equation modelling was conducted using SmartPLS version 4 (SmartPLS GmbH, Oststeinbek, Germany).

### Study setting

The study was conducted within the Makkah Health Cluster, Saudi Arabia, one of the integrated healthcare networks established as part of the national healthcare transformation under Saudi Vision 2030. The cluster comprises tertiary and secondary healthcare facilities providing services across multiple clinical specialties. This setting provided an appropriate organizational context for examining the relationships among leadership, organizational culture, knowledge sharing, and nursing innovation.

### Participants and sampling

The target population comprised registered nurses employed within the Makkah Health Cluster who were directly involved in clinical care. According to institutional workforce records, the target population consisted of approximately 4,425 registered nurses working across emergency departments, intensive care units, medical and surgical wards, outpatient departments, and other clinical units.

A proportionate stratified random sampling technique was employed to ensure representation of nurses across participating hospitals and clinical departments. The participating hospitals and major clinical departments constituted the sampling strata, and the number of nurses allocated to each stratum was determined proportionally according to its relative representation within the nursing workforce. Within each stratum, eligible nurses were selected from the available nursing staff lists using random selection procedures. This approach was intended to reduce selection bias and improve representation of nurses across different clinical settings within the Makkah Health Cluster.

### Sample size and participant flow

The minimum required sample size was estimated using Cochran’s formula with finite population correction because the target population of registered nurses within the Makkah Health Cluster was known (N = 4,425). Assuming a 95% confidence level, a 5% margin of error, and a population proportion of 0.50, the initial Cochran estimate for an unrestricted population was approximately 384 participants. After applying the finite population correction for N = 4,425, the minimum required sample size was approximately 354 participants.

An *a priori* statistical power analysis was additionally conducted using G*Power version 3.1 ( Faul et al., 2009) to assess the adequacy of the sample for the proposed structural relationships. Assuming a medium effect size (f ^2^ = 0.15), α = 0.05, statistical power of 0.95, and a maximum of three predictors for an endogenous construct, the estimated minimum sample size was 119 participants. The final analytical sample of 531 registered nurses therefore exceeded both the Cochran-based minimum sample requirement and the statistical power requirement for the proposed PLS-SEM analysis.

A total of 800 registered nurses were invited to participate in the study. Of these, 565 nurses submitted the questionnaire, yielding an overall response rate of 70.6%. Following eligibility verification and data screening, 34 questionnaires were excluded because they were incomplete or did not meet the predefined eligibility criteria. Consequently, 531 complete and valid questionnaires were retained for the final analysis, representing a usable response rate of 66.4% of the invited sample.

### Eligibility criteria

***Inclusion criteria***

Participants were eligible if they: were registered nurses employed within the Makkah Health Cluster; had worked for at least six months in their current organization; and were directly involved in clinical nursing services.

***Exclusion criteria***

Nurses were excluded if they were: undergoing internship or clinical training; on extended leave during the data collection period; or not actively involved in clinical practice.

### Study variables and measurements

Four constructs were assessed using standardized self-administered questionnaire items: transformational leadership, organizational culture, knowledge sharing, and nursing innovation. All items were rated using a five-point Likert response format.

***Transformational leadership***

Transformational leadership was measured using a 16-item questionnaire adapted from the Multifactor Leadership Questionnaire (MLQ) developed by Bass and Avolio. The instrument evaluates four dimensions: idealized influence, inspirational motivation, intellectual stimulation, and individualized consideration.

***Organizational culture***

Organizational culture was measured using a 10-item scale adapted from Cameron and Quinn (2011) assessing organizational values, collaboration, openness, innovation orientation, and adaptability.

***Knowledge sharing***

Knowledge sharing was measured using a 10-item instrument adapted from Bock et al. (2005) assessing employees’ willingness to exchange information, professional experience, clinical knowledge, and expertise within formal and informal workplace interactions.

***Nursing innovation***

Nursing innovation was measured using a 10-item instrument adapted from Scott and Bruce (1994) assessing innovative work behaviour, including idea generation, idea promotion, and implementation of innovative practices within clinical settings.

### Data collection

Data were collected through an anonymous online questionnaire administered using Google Forms. Before accessing the questionnaire, eligible nurses were provided with an electronic participant information sheet explaining the purpose of the study, study procedures, voluntary participation, confidentiality, and their rights as research participants. Electronic written informed consent was obtained by requiring participants to indicate their agreement before proceeding to the questionnaire. The survey included validated instruments assessing transformational leadership, organizational culture, knowledge sharing, and nursing innovation, together with demographic information such as age, sex, educational level, years of professional experience, and clinical department.

To minimize information bias, all questionnaires were completed anonymously using standardized self-administered instruments. Participants were informed that they could discontinue their participation at any point without consequences. Questionnaires containing incomplete responses were excluded from the analysis, and all study data were securely stored and used solely for research purposes.

### Statistical analysis

Data were analysed using IBM SPSS Statistics version 26 and SmartPLS version 4. Descriptive statistics were used to summarize participant characteristics and the distributions of the study variables.

The measurement model was evaluated following PLS-SEM procedures. Indicator reliability was assessed using outer loadings. Internal consistency reliability was evaluated using Cronbach’s alpha, rho_A, and composite reliability (CR), while convergent validity was evaluated using average variance extracted (AVE). Values ≥0.70 for indicator loadings and reliability coefficients and ≥ 0.50 for AVE were considered indicative of satisfactory measurement properties.

Discriminant validity was assessed using the Fornell–Larcker criterion. The structural model was subsequently evaluated using standardized path coefficients (β), coefficients of determination (R ^2^ and adjusted R ^2^), effect sizes (f ^2^), and predictive relevance using Q ^2^_predict. Predictive performance was additionally examined using root mean square error (RMSE) and mean absolute error (MAE).

The statistical significance of the hypothesized structural relationships was assessed using non-parametric bootstrapping with 5,000 resamples. Standardized path coefficients, t-statistics, and p-values were examined for the direct structural relationships. The mediating role of knowledge sharing was assessed using specific indirect effects, with the significance of the indirect pathways evaluated using the corresponding bootstrapped t-statistics and p-values. Statistical significance was established at p < 0.05.

## Results

### Participant characteristics

A total of 531 registered nurses participated in this study. As presented in Table 1, most respondents were female (71.8%), aged 26–35 years (44.8%), and of Saudi nationality (63.8%). More than half held a bachelor’s degree (52.4%), while staff nurses constituted the largest professional group (70.1%). The Medical/Surgical department represented the highest proportion of respondents (40.3%), and the largest proportion of participants had 4–5 years of clinical experience (25.8%). The respondents were recruited from six hospitals within the Makkah Health Cluster, with the largest proportion coming from Noor Specialist Hospital (27.3%).

**Table 1.** Demographic characteristics of the respondents (N = 531).

Characteristic	Category	n (%)
Age	26–35 years	238 (44.8)
	36–45 years	191 (36.0)
	≥46 years	102 (19.2)
Sex	Male	150 (28.2)
	Female	381 (71.8)
Nationality	Saudi	339 (63.8)
	Non-Saudi	192 (36.2)
Education level	Diploma	139 (26.2)
	Bachelor’s degree	278 (52.4)
	Master’s degree	108 (20.3)
	Doctoral degree	6 (1.1)
Professional position	Staff nurse	372 (70.1)
	Charge nurse	62 (11.7)
	Nurse manager	48 (9.0)
	Nurse educator	29 (5.5)
	Other	20 (3.8)
Department	Emergency	78 (14.7)
	Intensive Care Unit (ICU)	101 (19.0)
	Medical/Surgical	214 (40.3)
	Outpatient	79 (14.9)
	Other	59 (11.1)
Years of experience	1 year	55 (10.4)
	2–3 years	128 (24.1)
	4–5 years	137 (25.8)
	6–7 years	114 (21.5)
	≥8 years	97 (18.3)
Hospital	King Abdullah Medical City (KAMC)	108 (20.3)
	Noor Specialist Hospital (NSH)	145 (27.3)
	Maternity and Child Hospital (MCH)	97 (18.3)
	King Abdulaziz Hospital (KAH)	63 (11.9)
	Hiraa General Hospital (HGH)	60 (11.3)
	King Faisal Hospital (KFH)	58 (10.9)

### Descriptive statistics

The descriptive analysis indicated generally positive perceptions across all study constructs. The mean total scores were 60.61 (SD = 10.22) for transformational leadership, 37.79 (SD = 6.46) for organizational culture, 37.83 (SD = 6.43) for knowledge sharing, and 37.75 (SD = 6.22) for nursing innovation ( Table 2).

**Table 2.** Descriptive statistics of study variables.

Construct	Mean	SD
Transformational Leadership	60.61	10.22
Organizational Culture	37.79	6.46
Knowledge Sharing	37.83	6.43
Nursing Innovation	37.75	6.22

*Note*: SD = standard deviation.

### Measurement model assessment

The measurement model was assessed by examining indicator reliability, internal consistency reliability, and convergent validity. All indicator outer loadings exceeded the recommended threshold of 0.70, ranging from 0.714 to 0.846. Specifically, outer loadings ranged from 0.714 to 0.846 for transformational leadership, from 0.768 to 0.821 for organizational culture, from 0.747 to 0.831 for knowledge sharing, and from 0.742 to 0.826 for nursing innovation. No indicators were therefore removed from the measurement model.

Internal consistency reliability was satisfactory across all constructs. Cronbach’s alpha values ranged from 0.926 to 0.959, while composite reliability (rho_C) values ranged from 0.938 to 0.963. The rho_A coefficients were similarly satisfactory, ranging from 0.927 to 0.959. Convergent validity was also established, as the average variance extracted (AVE) exceeded the recommended threshold of 0.50 for all constructs, ranging from 0.602 to 0.643. Collectively, these findings demonstrate satisfactory indicator reliability, internal consistency reliability, and convergent validity of the measurement model ( Table 3).

**Table 3.** Measurement model assessment.

Construct	Indicator	Outer loading	Cronbach’s α	rho_A	rho_C	AVE
Transformational Leadership	TL1	0.788	0.959	0.959	0.963	0.619
	TL2	0.714				
	TL3	0.796				
	TL4	0.759				
	TL5	0.811				
	TL6	0.823				
	TL7	0.784				
	TL8	0.781				
	TL9	0.769				
	TL10	0.846				
	TL11	0.807				
	TL12	0.753				
	TL13	0.821				
	TL14	0.750				
	TL15	0.769				
	TL16	0.805				
Organizational Culture	OC1	0.821	0.938	0.939	0.947	0.643
	OC2	0.821				
	OC3	0.790				
	OC4	0.783				
	OC5	0.783				
	OC6	0.801				
	OC7	0.768				
	OC8	0.813				
	OC9	0.818				
	OC10	0.818				
Knowledge Sharing	KS1	0.831	0.933	0.935	0.943	0.625
	KS2	0.802				
	KS3	0.747				
	KS4	0.763				
	KS5	0.826				
	KS6	0.760				
	KS7	0.748				
	KS8	0.795				
	KS9	0.799				
	KS10	0.825				
Nursing Innovation	NI1	0.751	0.926	0.927	0.938	0.602
	NI2	0.805				
	NI3	0.748				
	NI4	0.776				
	NI5	0.742				
	NI6	0.784				
	NI7	0.779				
	NI8	0.766				
	NI9	0.777				
	NI10	0.826				

### Discriminant validity

Discriminant validity was assessed using the Fornell–Larcker criterion. As shown in Table 4, the square root of the AVE for each construct exceeded its correlations with all other constructs. The diagonal values were 0.790 for knowledge sharing, 0.776 for nursing innovation, 0.802 for organizational culture, and 0.787 for transformational leadership. These values were consistently higher than the corresponding inter-construct correlations, which ranged from 0.483 to 0.737. Thus, the Fornell–Larcker criterion supported adequate discriminant validity among the four constructs.

**Table 4.** Fornell–Larcker criterion.

Construct	KS	NI	OC	TL
KS	0.790			
NI	0.730	0.776		
OC	0.616	0.613	0.802	
TL	0.737	0.688	0.483	0.787

### Structural model assessment

The structural model was evaluated by examining the coefficient of determination (R ^2^), adjusted R ^2^, effect size (f ^2^), and predictive relevance (Q ^2^_predict). The model demonstrated substantial explanatory power for both endogenous constructs. Transformational leadership and organizational culture jointly explained 63.1% of the variance in knowledge sharing (R ^2^ = 0.631; adjusted R ^2^ = 0.630). Furthermore, transformational leadership, organizational culture, and knowledge sharing collectively explained 62.0% of the variance in nursing innovation (R ^2^ = 0.620; adjusted R ^2^ = 0.618).

Effect-size analysis indicated that transformational leadership exerted the strongest effect on knowledge sharing (f ^2^ = 0.683), followed by organizational culture (f ^2^ = 0.240). For nursing innovation, the effect sizes were smaller but remained meaningful, with f ^2^ values of 0.118 for transformational leadership, 0.101 for organizational culture, and 0.116 for knowledge sharing. These findings indicate that transformational leadership was particularly influential in explaining knowledge-sharing behaviour, whereas nursing innovation was influenced by the combined contributions of transformational leadership, organizational culture, and knowledge sharing.

The model also demonstrated predictive relevance. The Q ^2^_predict values were positive for both knowledge sharing (Q ^2^_predict = 0.629) and nursing innovation (Q ^2^_predict = 0.573), indicating predictive capability for both endogenous constructs. The corresponding prediction errors were RMSE = 0.612 and MAE = 0.489 for knowledge sharing and RMSE = 0.656 and MAE = 0.529 for nursing innovation. Collectively, these results indicate satisfactory explanatory and predictive performance of the proposed structural model. Collectively, these results indicate satisfactory explanatory and predictive performance of the proposed structural model ( Table 5).

**Table 5.** Structural model assessment.

Endogenous construct/relationship	R ^2^	Adjusted R ^2^	f ^2^	Q ^2^_predict	RMSE	MAE
**Knowledge Sharing**	0.631	0.630		0.629	0.612	0.489
TL → Knowledge Sharing			0.683			
OC → Knowledge Sharing			0.240			
**Nursing Innovation**	0.620	0.618		0.573	0.656	0.529
TL → Nursing Innovation			0.118			
OC → Nursing Innovation			0.101			
KS → Nursing Innovation			0.116			

Notes: TL: transformational leadership; OC: organizational culture; KS: knowledge sharing; R ^2^: coefficient of determination; f ^2^: effect size; Q ^2^_predict: predictive relevance; RMSE: root mean square error; MAE: mean absolute error.

### Hypothesis testing

Bootstrapping with 5,000 resamples was used to examine the statistical significance of the hypothesized structural relationships. As presented in Table 6, all five direct hypotheses were supported. Transformational leadership had a significant positive effect on nursing innovation (β = 0.313, t = 8.114, p < 0.001), supporting H1. Transformational leadership also demonstrated a strong positive effect on knowledge sharing (β = 0.573, t = 20.896, p < 0.001), supporting H2.

**Table 6.** Direct effects and hypothesis testing.

Hypothesis	Structural path	β	SD	t-value	p-value	Decision
H1	Transformational Leadership → Nursing Innovation	0.313	0.039	8.114	<0.001	Supported
H2	Transformational Leadership → Knowledge Sharing	0.573	0.027	20.896	<0.001	Supported
H3	Organizational Culture → Nursing Innovation	0.249	0.033	7.625	<0.001	Supported
H4	Organizational Culture → Knowledge Sharing	0.340	0.031	11.048	<0.001	Supported
H5	Knowledge Sharing → Nursing Innovation	0.345	0.042	8.164	<0.001	Supported

Organizational culture had a significant positive effect on nursing innovation (β = 0.249, t = 7.625, p < 0.001), supporting H3, and on knowledge sharing (β = 0.340, t = 11.048, p < 0.001), supporting H4. Finally, knowledge sharing significantly and positively influenced nursing innovation (β = 0.345, t = 8.164, p < 0.001), supporting H5. Among the direct relationships, transformational leadership demonstrated the strongest effect on knowledge sharing.

### Mediation analysis

Knowledge sharing significantly mediated the relationships between transformational leadership and nursing innovation and between organizational culture and nursing innovation. The specific indirect effect of transformational leadership on nursing innovation through knowledge sharing was positive and statistically significant (β = 0.198, t = 7.726, p < 0.001), supporting H6. Similarly, the indirect effect of organizational culture on nursing innovation through knowledge sharing was significant (β = 0.117, t = 6.434, p < 0.001), supporting H7 ( Table 7).

**Table 7.** Indirect effects and mediation analysis.

Hypothesis	Indirect pathway	Indirect β	t-value	p-value	Mediation	Decision
H6	TL → KS → NI	0.198	7.726	<0.001	Complementary partial mediation	Supported
H7	OC → KS → NI	0.117	6.434	<0.001	Complementary partial mediation	Supported

Because the corresponding direct effects of transformational leadership on nursing innovation (β = 0.313, p < 0.001) and organizational culture on nursing innovation (β = 0.249, p < 0.001) remained statistically significant in the presence of the mediator, and the direct and indirect effects operated in the same positive direction, knowledge sharing demonstrated complementary partial mediation in both relationships. These findings indicate that transformational leadership and organizational culture contribute to nursing innovation both directly and indirectly through enhanced knowledge-sharing behaviours.

## Discussion

This study examined the relationships among transformational leadership, organizational culture, knowledge sharing, and nursing innovation among registered nurses in the Makkah Health Cluster, Saudi Arabia. The findings provide empirical support for the proposed conceptual model, demonstrating that transformational leadership and organizational culture were associated with nursing innovation both directly and indirectly through knowledge sharing. All direct relationships were statistically significant, while knowledge sharing demonstrated complementary partial mediation in the relationships between transformational leadership and nursing innovation and between organizational culture and nursing innovation. Collectively, these findings suggest that nursing innovation is associated with an interplay of leadership, organizational context, and processes through which professional knowledge is exchanged and applied.

A particularly notable finding was the strong relationship between transformational leadership and knowledge sharing (β = 0.573), which represented the largest direct path in the structural model. Transformational leadership was also directly associated with nursing innovation (β = 0.313). These findings indicate that transformational leaders may promote innovation not only by directly encouraging nurses to challenge established practices and develop new approaches, but also by creating conditions in which professional knowledge can circulate more effectively among staff. Previous research has similarly linked transformational leadership with creativity, employee participation, organizational commitment, and innovative behaviour ( Boamah et al., 2019; Hughes et al., 2018; Bagheri, 2020). However, the comparatively stronger association with knowledge sharing observed in the present study suggests that the influence of transformational leadership on innovation may depend substantially on its capacity to stimulate interpersonal and organizational knowledge exchange.

This pattern can be interpreted through Social Exchange Theory (SET). Leaders who provide intellectual stimulation, individualized support, and a compelling organizational vision may create relational conditions characterized by trust, reciprocity, and psychological willingness to contribute beyond formally prescribed duties. Knowledge sharing is particularly dependent on such conditions because professional knowledge and clinical experience are not always transferred automatically through formal organizational structures. Nurses must be willing to communicate experiences, exchange clinical insights, and contribute their expertise to colleagues. The present findings therefore suggest that transformational leadership may create a social environment in which nurses reciprocate supportive leadership through greater participation in knowledge-sharing and innovative activities.

Organizational culture was also positively associated with both knowledge sharing (β = 0.340) and nursing innovation (β = 0.249). These findings reinforce the importance of organizational context in determining whether innovative behaviour can develop and be sustained. Cultures characterized by collaboration, openness, adaptability, and learning may reduce organizational barriers to exchanging ideas and experimenting with alternative approaches to clinical practice. This interpretation is consistent with previous evidence linking innovation-oriented organizational cultures with organizational learning, interdisciplinary collaboration, and employees’ readiness for change ( Hartmann & Herb, 2020; Naranjo-Valencia et al., 2021; Shujahat et al., 2021). Importantly, the present findings indicate that organizational culture is associated not merely with innovation as an outcome but also with the knowledge-sharing processes through which innovation can develop.

The simultaneous effects of transformational leadership and organizational culture are theoretically important. Leadership and culture are sometimes examined as independent antecedents of innovation; however, the present findings suggest that they represent complementary organizational resources. Leadership may provide interpersonal motivation and direction, whereas organizational culture establishes the broader norms and expectations governing collaboration, learning, and knowledge exchange. The considerable variance explained in knowledge sharing (R ^2^ = 0.631) indicates that these organizational conditions collectively account for a considerable proportion of differences in nurses’ knowledge-sharing behaviour. Thus, interventions aimed solely at developing individual leaders without addressing the surrounding organizational culture, or vice versa, may not fully capitalize on their potential contribution to organizational learning and innovation.

Knowledge sharing itself demonstrated a significant positive relationship with nursing innovation (β = 0.345). This finding is particularly relevant in nursing environments, where knowledge is distributed across professionals with different clinical experiences, specialties, and responsibilities. Exchange of clinical expertise and professional experience can enable nurses to combine existing knowledge, identify deficiencies in established practices, and develop or implement alternative solutions. Previous research has similarly suggested that knowledge-sharing processes facilitate collective problem-solving, organizational learning, and innovation ( Nguyen et al., 2021; Islam et al., 2022). The present findings extend this evidence by demonstrating that knowledge sharing functions both as an independent predictor of nursing innovation and as a mechanism connecting broader organizational resources with innovative nursing behaviour.

The mediation findings provide further insight into this mechanism. Knowledge sharing significantly mediated the relationship between transformational leadership and nursing innovation (indirect β = 0.198, p < 0.001) and the relationship between organizational culture and nursing innovation (indirect β = 0.117, p < 0.001). Because the corresponding direct relationships remained significant and operated in the same positive direction, both pathways represented complementary partial mediation. Thus, transformational leadership and supportive organizational culture appear to be associated with nursing innovation partly through their relationships with the exchange and application of professional knowledge. Other mechanisms, such as psychological empowerment, intrinsic motivation, organizational commitment, innovation climate, or perceived organizational support, may also contribute to these relationships and warrant further investigation.

These mediation findings are consistent with the Knowledge-Based View (KBV), which conceptualizes knowledge as a strategically important organizational resource whose value depends on its integration, transfer, and application. Within healthcare organizations, professional knowledge is often highly specialized and dispersed among individuals and clinical units. Leadership and organizational culture may create conditions conducive to knowledge exchange, but innovation is more likely to emerge when that knowledge is actively shared and recombined to address clinical and organizational challenges. The findings are also consistent with Organizational Learning Theory, which emphasizes knowledge acquisition, dissemination, interpretation, and application as central processes underlying organizational adaptation and innovation. Together with SET, these perspectives provide complementary explanations of how organizational structures and interpersonal processes jointly contribute to nursing innovation.

The explanatory and predictive performance of the structural model further supports the relevance of this integrated perspective. Transformational leadership and organizational culture explained 63.1% of the variance in knowledge sharing, while transformational leadership, organizational culture, and knowledge sharing jointly explained 62.0% of the variance in nursing innovation. Positive Q ^2^_predict values for knowledge sharing (0.629) and nursing innovation (0.573) further indicated predictive relevance. These findings suggest that the proposed model accounts for a considerable proportion of variation in knowledge sharing and innovative behaviour among nurses, while also leaving room for additional individual, team-level, and organizational determinants.

Although the present findings generally align with previous research, the literature has not consistently established how leadership and organizational culture translate into innovative behaviour in healthcare settings. Prior studies have often examined direct associations between organizational antecedents and innovation, while the processes connecting these organizational conditions to nurses’ innovative behaviour remain comparatively less developed. The present findings help address this limitation by showing that knowledge sharing is associated with nursing innovation while simultaneously serving as a complementary pathway linking transformational leadership and organizational culture with innovation. Importantly, the persistence of significant direct relationships after accounting for knowledge sharing indicates that knowledge exchange represents only one part of a broader organizational process, suggesting that additional psychological and contextual mechanisms should be considered in future models.

The study contributes to the literature in several ways. First, rather than examining transformational leadership, organizational culture, knowledge sharing, and innovation as isolated relationships, it integrates these constructs within a single structural model. Second, it identifies knowledge sharing as a complementary mechanism through which both leadership and organizational culture contribute to nursing innovation. Third, the findings provide empirical evidence from the Makkah Health Cluster, extending research on organizational determinants of nursing innovation to the context of an integrated healthcare system undergoing substantial transformation in Saudi Arabia. The results therefore add contextual evidence to the application of SET, KBV, and Organizational Learning Theory in healthcare organizations and highlight the importance of considering both structural and relational conditions when seeking to strengthen innovation.

### Strengths and limitations

This study has several strengths. First, it examined leadership, organizational, and knowledge-management determinants simultaneously using PLS-SEM, enabling the assessment of both direct and indirect relationships within a theoretically integrated model. Second, the measurement model demonstrated satisfactory indicator reliability, internal consistency reliability, convergent validity, and discriminant validity. Third, the relatively large analytical sample of 531 registered nurses included participants from multiple hospitals and clinical departments within the Makkah Health Cluster, providing representation across different nursing practice environments. Finally, the assessment of both explanatory and predictive performance provided information beyond statistical significance alone.

Several limitations should nevertheless be considered. First, the cross-sectional design prevents determination of temporal ordering and causal relationships; therefore, the structural paths should be interpreted as statistical associations rather than evidence of causality. Second, although participants were recruited from multiple hospitals, all institutions belonged to a single healthcare cluster in Saudi Arabia, which may limit the generalizability of the findings to healthcare organizations operating under different institutional, cultural, or regulatory conditions. Third, all constructs were assessed using self-administered questionnaires, making the findings potentially susceptible to self-report bias, social desirability bias, and common method variance. Finally, although the model explained considerable variance in knowledge sharing and nursing innovation, other potentially relevant determinants, including psychological empowerment, innovation climate, organizational support, staffing conditions, and individual readiness for change, were not incorporated.

### Implications for practice

The findings have several implications for nursing management and healthcare administration. Leadership development initiatives should extend beyond conventional supervisory competencies and strengthen behaviours associated with intellectual stimulation, individualized support, collaborative decision-making, and encouragement of new ideas. However, leadership development alone may be insufficient. Healthcare organizations should simultaneously cultivate organizational cultures that support openness, interdisciplinary collaboration, continuous learning, and constructive experimentation.

Knowledge-sharing infrastructure also warrants explicit managerial attention, given the significant role of knowledge sharing in the relationships between leadership, organizational culture, and nursing innovation observed in this study. Hospitals may facilitate knowledge exchange through interdisciplinary case discussions, structured mentoring, communities of practice, peer-learning programmes, clinical innovation forums, and accessible digital platforms for exchanging professional knowledge and practice-based experience. Within the context of Saudi healthcare transformation, integrating leadership development, supportive organizational culture, and knowledge-management strategies may strengthen organizational conditions conducive to nursing innovation and continuous quality improvement.

### Future research

Future studies should employ longitudinal or prospective designs to examine the temporal relationships among transformational leadership, organizational culture, knowledge sharing, and nursing innovation. Multi-centre investigations involving healthcare clusters and institutions across different regions of Saudi Arabia and other countries would help establish the generalizability of the proposed model. Future research should also investigate additional mechanisms that may operate alongside knowledge sharing, including psychological empowerment, innovation climate, perceived organizational support, organizational commitment, employee resilience, and work engagement. Multilevel approaches could further distinguish individual-, team-, and organizational-level influences on nursing innovation. Finally, qualitative and mixed-methods research may provide deeper insight into how nurses experience leadership, organizational culture, and knowledge exchange and how these experiences facilitate or constrain innovation in everyday clinical practice.

## Conclusion

This study identified significant relationships among transformational leadership, organizational culture, knowledge sharing, and nursing innovation among registered nurses within the Makkah Health Cluster, Saudi Arabia. Transformational leadership and organizational culture were positively associated with both knowledge sharing and nursing innovation, while knowledge sharing was positively associated with nursing innovation and demonstrated complementary partial mediation in both relationships. These findings support an integrated perspective in which leadership, organizational context, and knowledge-sharing processes are jointly associated with innovative nursing behaviour.

The findings also underscore the practical relevance of creating organizational conditions that support transformational leadership, collaborative cultures, and effective knowledge exchange. Healthcare organizations may therefore benefit from integrating leadership development with organizational and knowledge-management strategies that encourage collaboration, learning, and the exchange of clinical expertise. Such strategies may strengthen conditions conducive to nursing innovation and support continuous quality improvement within healthcare systems undergoing transformation, including the Saudi healthcare system under Vision 2030.

## Ethical considerations

Ethical approval for this study was granted by the Research Ethics Committee (Institutional Review Board) of Lincoln University College, Malaysia (Approval No. LUC/MKT/SP/017/223). Prior to participation, all eligible nurses provided electronic written informed consent. Participation was entirely voluntary, participants could withdraw from the study at any stage without penalty, and strict confidentiality was maintained throughout the research process. No personally identifiable information was collected or retained.

## STROBE checklist

Data are available under the terms of the Creative Commons Attribution 4.0 International (CC BY 4.0) licence.

## Data Availability

The data supporting the findings of this study consist of de-identified questionnaire responses collected from registered nurses working in hospitals within the Makkah Health Cluster, Saudi Arabia. These data have not been deposited in a public repository because they were collected from human participants under conditions of confidentiality and are subject to institutional and ethical restrictions established by the Research Ethics Committee. Under the approved research protocol, participant-level data cannot be released publicly in order to safeguard participant privacy and confidentiality. Researchers interested in accessing the de-identified dataset for legitimate academic or non-commercial research may submit a written request to the corresponding author at khadijalafialjarary@gmail.com. Requests will be evaluated on a case-by-case basis and may require approval from the Research Ethics Committee of Lincoln University College and the participating healthcare institution. Access will be granted only when the proposed use is consistent with the approved ethical protocol and adequate measures are in place to protect participant confidentiality. Zenodo. *Questionnaire for “The Mediating Role of Knowledge Sharing in the Relationship Between Transformational Leadership, Organizational Culture, and Nursing Innovation in the Makkah Health Cluster”* [Data set]. Zenodo. https://doi.org/10.5281/zenodo.21144008 ( Aljarary, K. L., & Hassan, H. C. 2026). This project contains the following underlying data:
• Questionnaire.pdf (Questionnaire containing measurement items for transformational leadership, organizational culture, knowledge sharing, nursing innovation, and demographic characteristics.) Data are available under the terms of the Creative Commons Attribution 4.0 International (CC BY 4.0) licence. This study was reported in accordance with the Strengthening the Reporting of Observational Studies in Epidemiology (STROBE) Statement. Zenodo. The Mediating Role of Knowledge Sharing in the Relationship Between Transformational Leadership, Organizational Culture, and Nursing Innovation in the Makkah Health Cluster: Strobe Checklist [Data set]. Zenodo. https://doi.org/10.5281/zenodo.21099279 ( Khadija Lafi, A., & Hassan, H. C. 2026).
