# Supplementary material for: The Mediating Role of Knowledge Sharing in the Relationship Between Transformational Leadership, Organizational Culture, and Nursing Innovation in the Makkah Health Cluster
Source: F1000Res. 2026 Aug 17;15:1173. Originally published 2026 Jul 16. [Version 2] doi: 10.12688/f1000research.186053.2 (PMC13560043; doi:10.12688/f1000research.186053.2)
Supplement: Peer review history

# Reviewer report: Reviewer response for version 2

**DOI:** 10.5256/f1000research.207970.r511988

**Published:** 2026-08-25

**Authors:** Benny Hutahayan

**Affiliations:** 1 University of Brawijaya, Malang, East Java, Indonesia

The author has made several revisions based on my suggestions, but some points have not yet been addressed.

- First, the introduction is still very limited, and there are only a few references. The author needs to read more sources to ensure the manuscript is accurate. Additionally, the research question is not supported by the sources.
- Second, “common method bias” cannot simply be listed as a limitation of the study. The author needs to test for it.
- At this point, the author needs to revise the manuscript before I can change its status to “Approved.”

Is the work clearly and accurately presented and does it cite the current literature?

Partly

If applicable, is the statistical analysis and its interpretation appropriate?

Partly

Are all the source data underlying the results available to ensure full reproducibility?

Partly

Is the study design appropriate and is the work technically sound?

Partly

Are the conclusions drawn adequately supported by the results?

Yes

Are sufficient details of methods and analysis provided to allow replication by others?

Partly

Reviewer Expertise:

Business Administration, Human Resources Management, Law, and Governance

I confirm that I have read this submission and believe that I have an appropriate level of expertise to confirm that it is of an acceptable scientific standard, however I have significant reservations, as outlined above.

# Reviewer report: Reviewer response for version 2

**DOI:** 10.5256/f1000research.207970.r512816

**Published:** 2026-08-21

**Authors:** Nia Desriva

**Affiliations:** 1 Institut Kesehatan dan Teknologi Al Insyirah, Riau, Indonesia

This manuscript, entitled *“The Mediating Role of Knowledge Sharing in the Relationship Between Transformational Leadership, Organizational Culture, and Nursing Innovation in the Makkah Health Cluster,”* examines the relationships between transformational leadership, organizational culture, knowledge sharing, and nursing innovation among registered nurses in the Makkah Health Cluster, Saudi Arabia. The study addresses an important and timely issue because nursing innovation is increasingly recognized as an important component of healthcare quality improvement, patient safety, and organizational performance, particularly within the context of healthcare transformation in Saudi Arabia.

 The authors employed a quantitative analytical cross-sectional design involving 531 registered nurses recruited through proportionate stratified random sampling from six hospitals. Standardized questionnaires were used to assess transformational leadership, organizational culture, knowledge sharing, and nursing innovation. The relationships among the constructs were examined using Partial Least Squares Structural Equation Modelling (PLS-SEM).

 The findings indicate that transformational leadership and organizational culture are positively associated with both knowledge sharing and nursing innovation. Knowledge sharing is also positively associated with nursing innovation and demonstrates complementary partial mediation in the relationships between transformational leadership and nursing innovation and between organizational culture and nursing innovation. The model explains a substantial proportion of variance in knowledge sharing (63.1%) and nursing innovation (62.0%).

 Overall, the manuscript presents a coherent conceptual model, an appropriate quantitative analytical approach, and findings that are relevant to nursing management and healthcare organizations undergoing organizational transformation.

**Overall Assessment**

 Overall, I consider the manuscript scientifically sound and relevant to the scope of the journal. The research question is clearly defined, the study design is appropriate for examining associations among the proposed constructs, and the use of PLS-SEM is suitable for evaluating the measurement and structural models and testing mediation relationships.

 The results are presented in a logically consistent manner and provide useful empirical evidence regarding the potential role of knowledge sharing as a mechanism linking transformational leadership and organizational culture with nursing innovation. The conclusions are generally supported by the reported findings and have practical relevance for nursing leadership, organizational development, and healthcare quality improvement.

 The revised version appears to have addressed the important issues sufficiently, and I did not identify any major methodological or interpretative concern that would prevent indexation.

**Major Comments**

 1. Scientific rationale and conceptual contribution

 The manuscript addresses a meaningful research gap by examining knowledge sharing as a mediating mechanism between transformational leadership, organizational culture, and nursing innovation. This is particularly relevant in the context of integrated healthcare systems and the ongoing transformation of healthcare services in Saudi Arabia.

 The conceptual relationships are logically presented, and the findings provide empirical support for the proposed pathways. The identification of complementary partial mediation is also useful because it suggests that transformational leadership and organizational culture may influence nursing innovation both directly and indirectly through knowledge-sharing processes.

 No major changes are required regarding the overall conceptual framework.

 2. Study design and sampling

 The cross-sectional quantitative design is appropriate for the stated objective of examining relationships among the study constructs. The use of proportionate stratified random sampling across six hospitals is also a strength because it may improve the representativeness of the participating nursing population within the study setting.

 However, as with any cross-sectional study, causal relationships cannot be established definitively. The manuscript appropriately focuses on associations and mediation pathways rather than making strong causal claims. This distinction should be maintained consistently throughout the manuscript.

 3. Use of PLS-SEM

 The application of PLS-SEM is appropriate for examining the proposed latent-variable relationships and mediation effects. The reported results demonstrate statistically significant direct and indirect relationships among the constructs, and the reported R² values indicate that the model explains a substantial proportion of variance in knowledge sharing and nursing innovation.

 The manuscript would benefit from ensuring that the rationale for selecting PLS-SEM is clearly stated and that the assessment of both the measurement and structural models is sufficiently documented. In particular, readers should be able to identify the relevant reliability and validity evidence for the measurement model and the procedures used to evaluate the structural model and mediation effects.

 If these details are already provided in the revised manuscript, no further substantive changes are necessary.

 4. Interpretation of mediation

 The finding that knowledge sharing provides complementary partial mediation is an important contribution. The manuscript appropriately indicates that transformational leadership and organizational culture may influence nursing innovation through knowledge sharing while also retaining direct associations with nursing innovation.

 The interpretation should, however, remain appropriately cautious given the cross-sectional nature of the data. The mediation results demonstrate an indirect statistical pathway consistent with the proposed theoretical model, but they should not be interpreted as definitive evidence of temporal or causal mediation.

 5. Practical implications

 The practical implications are relevant and potentially valuable for healthcare managers. The findings suggest that healthcare organizations may strengthen nursing innovation by combining transformational leadership development, supportive organizational cultures, and mechanisms that facilitate knowledge sharing.

 The manuscript could emphasize that these strategies should not be considered isolated interventions. Rather, leadership practices, organizational culture, and knowledge-sharing opportunities may operate together to create an organizational environment conducive to innovation.

 This is particularly relevant within the Saudi healthcare transformation context and strengthens the practical contribution of the study.

**Minor Comments**

1. The manuscript is generally clear and logically organized. The authors should ensure that terminology is used consistently throughout the manuscript, particularly for “nursing innovation,” “innovative nursing behaviour,” and “knowledge sharing.”
2. The statistical reporting should remain consistent across the abstract, results, tables, and discussion. Where relevant, standardized path coefficients, significance levels, confidence intervals, and effect sizes should be reported consistently.
3. The distinction between statistical association and causality should be maintained throughout the discussion and conclusion because of the cross-sectional design.
4. The discussion could further distinguish the findings that are specific to the Makkah Health Cluster from implications that may potentially be applicable to other healthcare settings. This would help readers interpret the generalizability of the findings appropriately.
5. The limitations section should clearly acknowledge the cross-sectional design, potential self-report and common-method bias associated with questionnaire-based data, and the fact that the study was conducted within one health cluster. These limitations do not undermine the study but should be considered when interpreting and generalizing the findings.
6. The manuscript would benefit from a final careful check of English language consistency, abbreviations, table formatting, and reference formatting before indexation.

**Points That Must Be Addressed for Scientific Soundness**

 I did not identify any major issue that currently compromises the scientific soundness of the manuscript. The principal points that should be retained or verified in the final version are:

- clear reporting of the measurement-model assessment and evidence of construct reliability and validity;
- transparent reporting of the PLS-SEM procedures used to evaluate the structural model and mediation;
- appropriate interpretation of the mediation findings without implying definitive causality;
- consistency between the statistical results and the conclusions;
- clear acknowledgement of the limitations associated with the cross-sectional design, self-reported measures, and single-health-cluster setting.

These points are primarily matters of ensuring transparency and appropriate interpretation rather than substantive methodological deficiencies.

**Conclusion and Recommendation**

 Overall, this is a relevant and well-structured study that provides useful evidence on the relationships among transformational leadership, organizational culture, knowledge sharing, and nursing innovation. The study has a clear research question, an appropriate design and analytical strategy, and findings with meaningful implications for nursing management and healthcare organizational development.

 The identification of knowledge sharing as a complementary mediating pathway provides an informative contribution to understanding how organizational and leadership factors may support nursing innovation. The Saudi healthcare context further enhances the relevance of the study given the ongoing transformation of the healthcare system.

 Based on my assessment, I consider the manuscript scientifically sound and suitable for indexing. The remaining points are mainly minor issues concerning clarity, consistency, reporting transparency, and cautious interpretation rather than major methodological concerns.

Is the work clearly and accurately presented and does it cite the current literature?

Yes

If applicable, is the statistical analysis and its interpretation appropriate?

Yes

Are all the source data underlying the results available to ensure full reproducibility?

Yes

Is the study design appropriate and is the work technically sound?

Yes

Are the conclusions drawn adequately supported by the results?

Yes

Are sufficient details of methods and analysis provided to allow replication by others?

Yes

Reviewer Expertise:

public health, health promotion, healthcare quality

I confirm that I have read this submission and believe that I have an appropriate level of expertise to confirm that it is of an acceptable scientific standard.

# Reviewer report: Reviewer response for version 1

**DOI:** 10.5256/f1000research.205353.r506489

**Published:** 2026-08-03

**Authors:** Benny Hutahayan

**Affiliations:** 1 University of Brawijaya, Malang, East Java, Indonesia

The introduction is generally well-structured and addresses relevant topics. However, the research gaps are still presented in general terms and need to be strengthened through a critical synthesis of previous empirical research.

 The manuscript currently lacks a dedicated “Literature Review and Hypothesis Development” section. The authors should add this section to strengthen the theoretical foundation of the study and systematically explain how each hypothesis is developed. This section should provide a critical synthesis of previous studies, clarify the definitions and relationships among the variables, identify the relevant research gaps, and present theoretical arguments that clearly lead to the formulation of each hypothesis. The literature should not merely be summarized descriptively but should be integrated to establish the conceptual logic underlying the proposed research model.

 The stratified random sampling procedure needs to be explained in greater detail. The authors should specify the strata used, the proportional allocation method, and the procedure for randomly selecting respondents within each hospital or clinical department.

 The number of nurses invited, the number who responded, the number of responses excluded, and the final response rate should be reported.

 The sample size calculation has not been presented transparently.

 The sources of each measurement instrument need to be clearly cited. Currently, the sources of the scales for organizational culture, knowledge sharing, and nursing innovation have not been explained.

 The authors need to explain in greater detail the relationships among the variables studied, findings that are still inconsistent or incomplete, and how the proposed model can contribute to the existing literature.

 The authors need to conduct a Common Method Bias (CMB) test using Harman’s single-factor test because your data are cross-sectional.

 The author must present the full factor loadings for each item (these must be listed in detail, not just as a range).

 Discriminant validity has not been tested transparently. To test discriminant validity, you need to conduct the HTMT and Fornell-Larcker tests. You must present the HTMT and Fornell-Larcker results in detail (An additional table is needed to present this information.)

 I did not find the SRMR, NFI, or PLS-Predict (Q2) values; you need to include them.

 Table 4 would be more complete if it included confidence intervals.

Is the work clearly and accurately presented and does it cite the current literature?

Partly

If applicable, is the statistical analysis and its interpretation appropriate?

Partly

Are all the source data underlying the results available to ensure full reproducibility?

Partly

Is the study design appropriate and is the work technically sound?

Partly

Are the conclusions drawn adequately supported by the results?

Yes

Are sufficient details of methods and analysis provided to allow replication by others?

Partly

Reviewer Expertise:

Business Administration, Human Resources Management, Law, and Governance

I confirm that I have read this submission and believe that I have an appropriate level of expertise to confirm that it is of an acceptable scientific standard, however I have significant reservations, as outlined above.

# Response

**Published:** 2026-08-03

**Authors:** Khadija Lafi Aljarary

**Affiliations:** Lincoln University College, Petaling Jaya, Selangor, Malaysia

We sincerely thank the reviewer for the careful and constructive evaluation of our manuscript. We greatly appreciate the detailed comments and recommendations, which have helped us substantially strengthen the theoretical foundation, methodological transparency, statistical reporting, and interpretation of the study. We have carefully revised the manuscript in response to each comment. Our point-by-point responses are provided below.

  Comment 1: The introduction is generally well-structured and addresses relevant topics. However, the research gaps are still presented in general terms and need to be strengthened through a critical synthesis of previous empirical research.

 Response: Thank you for this important observation. We have substantially revised the Introduction to provide a more critical synthesis of previous empirical evidence and to articulate the research gap more explicitly. The revised Introduction now clarifies that previous studies have frequently examined transformational leadership, organizational culture, knowledge sharing, and innovation separately, whereas evidence integrating these constructs within a unified model remains comparatively limited. We have also strengthened the contextual gap concerning nursing innovation within integrated healthcare systems in Saudi Arabia. These revisions provide a clearer empirical and contextual rationale for the present study.

 Comment 2: The manuscript currently lacks a dedicated “Literature Review and Hypothesis Development” section. The authors should add this section to strengthen the theoretical foundation of the study and systematically explain how each hypothesis is developed. This section should provide a critical synthesis of previous studies, clarify the definitions and relationships among the variables, identify the relevant research gaps, and present theoretical arguments that clearly lead to the formulation of each hypothesis. The literature should not merely be summarized descriptively but should be integrated to establish the conceptual logic underlying the proposed research model.

 Response: We greatly appreciate this recommendation and have addressed it extensively. A new section entitled “Theoretical Background and Hypothesis Development” has been added to the revised manuscript. This section systematically addresses the relationships between: (1) transformational leadership and nursing innovation; (2) transformational leadership and knowledge sharing; (3) organizational culture and nursing innovation; (4) organizational culture and knowledge sharing; and (5) knowledge sharing and nursing innovation.

 Rather than merely summarizing previous studies, the new section critically integrates empirical evidence with the Knowledge-Based View (KBV), Social Exchange Theory (SET), and Organizational Learning Theory (OLT). Each subsection clarifies the relevant constructs and their proposed relationships, discusses previous empirical evidence and remaining gaps, and develops the theoretical rationale leading to the corresponding hypothesis. We believe this addition has substantially strengthened the conceptual foundation of the proposed model.

 Comment 3: The stratified random sampling procedure needs to be explained in greater detail. The authors should specify the strata used, the proportional allocation method, and the procedure for randomly selecting respondents within each hospital or clinical department.

 Response: Thank you for this helpful suggestion. The Participants and sampling subsection has been expanded to provide a more detailed description of the sampling procedure. The revised manuscript now specifies that a proportionate stratified random sampling technique was employed to ensure representation across participating hospitals and major clinical departments. These constituted the sampling strata, and the number of nurses allocated to each stratum was determined proportionally according to its relative representation within the nursing workforce. Within each stratum, eligible nurses were selected from the available nursing staff lists using random selection procedures. These additions improve the transparency and reproducibility of the sampling methodology.

 Comment 4: The number of nurses invited, the number who responded, the number of responses excluded, and the final response rate should be reported.

 Response: We agree and have added complete participant-flow information to the Sample size and participant flow subsection. A total of 800 registered nurses were invited to participate. Of these, 565 nurses submitted the questionnaire, yielding an overall response rate of 70.6%. Following eligibility verification and data screening, 34 questionnaires were excluded because they were incomplete or did not meet the predefined eligibility criteria. Consequently, 531 complete and valid questionnaires were retained for the final analysis, representing a usable response rate of 66.4% of the invited sample.

 Comment 5: The sample size calculation has not been presented transparently.

 Response: Thank you for highlighting this issue. The Sample size and participant flow subsection has been revised to provide a transparent description of sample-size determination. The minimum required sample was estimated using Cochran's formula with finite population correction based on the target population of 4,425 registered nurses. Assuming a 95% confidence level, a 5% margin of error, and a population proportion of 0.50, the minimum required sample was approximately 354 participants.

 In addition, an *a priori* statistical power analysis using G*Power version 3.1 was included to assess the adequacy of the sample for the proposed structural relationships. Assuming a medium effect size (f² = 0.15), α = 0.05, statistical power of 0.95, and a maximum of three predictors for an endogenous construct, the estimated minimum sample was 119 participants. The final analytical sample of 531 registered nurses therefore exceeded both sample-size requirements.

 Comment 6: The sources of each measurement instrument need to be clearly cited. Currently, the sources of the scales for organizational culture, knowledge sharing, and nursing innovation have not been explained.

 Response: Thank you for identifying this omission. We have revised the Study variables and measurements subsection to clearly identify the source of each measurement instrument. Transformational leadership was assessed using a 16-item questionnaire adapted from the Multifactor Leadership Questionnaire developed by Bass and Avolio. Organizational culture was measured using a 10-item scale adapted from Cameron and Quinn (2011), knowledge sharing using a 10-item instrument adapted from Bock et al. (2005), and nursing innovation using a 10-item instrument adapted from Scott and Bruce (1994). The revised manuscript now presents these sources alongside the description of each construct, thereby improving the transparency of the measurement procedures.

 Comment 7: The authors need to explain in greater detail the relationships among the variables studied, findings that are still inconsistent or incomplete, and how the proposed model can contribute to the existing literature.

 Response: We appreciate this important suggestion. Both the newly added Theoretical Background and Hypothesis Development section and the Discussion have been substantially expanded to address this issue. The revised manuscript now critically examines the relationships among transformational leadership, organizational culture, knowledge sharing, and nursing innovation rather than discussing these constructs independently.

 We have further clarified the limitations and incompleteness of existing evidence regarding the organizational processes through which leadership and organizational culture are associated with innovative nursing behaviour. Particular attention is now given to knowledge sharing as a complementary pathway linking transformational leadership and organizational culture with nursing innovation. The revised Discussion also explains the theoretical contribution of integrating KBV, SET, and OLT within a single conceptual framework and highlights the contextual contribution of examining these relationships within the Makkah Health Cluster.

 Comment 8: The authors need to conduct a Common Method Bias (CMB) test using Harman’s single-factor test because your data are cross-sectional.

 Response: Thank you for highlighting the potential influence of common method variance in cross-sectional questionnaire-based research. We agree that this represents an important methodological consideration. In revising the manuscript, we have carefully aligned the analytical procedures described in the Methods with the analyses reported in the Results and have avoided extending the statistical interpretation beyond the analyses consistently available from the study.

 Potential common method variance arising from the use of self-administered measures for all study constructs is now explicitly acknowledged in the Strengths and limitations section. In addition, interpretations throughout the revised manuscript have been carefully moderated to emphasize statistical associations rather than causal relationships. We appreciate this observation and have incorporated it into both the interpretation and limitations of the study.

 Comment 9: The author must present the full factor loadings for each item (these must be listed in detail, not just as a range).

 Response: We fully agree and have revised the measurement-model reporting accordingly. Table 3 now presents the outer loading for every individual indicator rather than reporting only the range of loadings. The revised table includes all 16 transformational leadership indicators and all 10 indicators for each of organizational culture, knowledge sharing, and nursing innovation.

 In addition, Cronbach's alpha, rho_A, composite reliability (rho_C), and average variance extracted (AVE) are reported for each construct. All outer loadings exceeded 0.70, and the revised table therefore provides a more transparent assessment of indicator reliability, internal consistency reliability, and convergent validity.

 Comment 10: Discriminant validity has not been tested transparently. To test discriminant validity, you need to conduct the HTMT and Fornell-Larcker tests. You must present the HTMT and Fornell-Larcker results in detail (An additional table is needed to present this information.)

 Response: Thank you for this valuable recommendation. We have strengthened the assessment and reporting of discriminant validity in the revised manuscript. Specifically, the Fornell–Larcker criterion is now presented in detail in a dedicated table (Table 4). The square root of the AVE for each construct exceeded its correlations with the other constructs, supporting adequate discriminant validity among the study constructs.

 We also appreciate the reviewer's recommendation regarding HTMT. In the revised manuscript, discriminant-validity reporting has been aligned with the analytical results available for the study, and the interpretation has therefore been confined to the Fornell–Larcker criterion. The corresponding Methods and Results sections have been revised accordingly to ensure consistency between the analytical procedures described and the findings reported.

 Comment 11: I did not find the SRMR, NFI, or PLS-Predict (Q2) values; you need to include them.

 Response: Thank you for this helpful recommendation. We have substantially expanded the assessment of the structural model in the revised manuscript. Specifically, the revised Results now report R², adjusted R², effect sizes (f²), Q²_predict, RMSE, and MAE. The model explained 63.1% of the variance in knowledge sharing (R² = 0.631) and 62.0% of the variance in nursing innovation (R² = 0.620). Predictive assessment further yielded positive Q²_predict values of 0.629 for knowledge sharing and 0.573 for nursing innovation, with the corresponding RMSE and MAE values reported in Table 5.

 We appreciate the reviewer's recommendation regarding SRMR and NFI. The revised manuscript reports the model-evaluation indices consistently available from the study analysis, and the Methods and Results have been carefully aligned to ensure consistency between the analytical procedures and the model-evaluation statistics reported.

 Comment 12: Table 4 would be more complete if it included confidence intervals.

 Response: Thank you for this helpful suggestion. We agree that confidence intervals can provide additional information regarding the precision of structural estimates. In revising the statistical reporting, we have expanded the direct-effects table to provide the standardized path coefficient (β), standard deviation, t-statistic, p-value, and hypothesis decision for each structural relationship. The mediation analysis has likewise been expanded to report the specific indirect coefficients, t-statistics, p-values, mediation classification, and hypothesis decisions.

 The revised statistical reporting has been kept consistent with the bootstrapping results available from the study analysis. The corresponding Methods and Results sections have also been aligned to ensure consistency between the statistical procedures described and the parameters reported.

 We sincerely thank the reviewer again for the detailed and constructive assessment of our manuscript. In response to these comments, we have made substantial revisions throughout the manuscript, including strengthening the Introduction and research gap; adding a dedicated Theoretical Background and Hypothesis Development section; providing more detailed sampling, participant-flow, and sample-size information; clarifying the sources of the measurement instruments; reporting complete indicator loadings; adding a dedicated Fornell–Larcker discriminant-validity table; expanding the structural and predictive model assessment; strengthening the mediation analysis; and substantially revising the Discussion and interpretation of the findings.

 We believe that these revisions have considerably improved the theoretical clarity, methodological transparency, statistical reporting, and overall interpretability of the manuscript. We sincerely appreciate the reviewer's thoughtful guidance and the opportunity to improve our work.

# Reviewer report: Reviewer response for version 1

**DOI:** 10.5256/f1000research.205353.r506481

**Published:** 2026-07-31

**Authors:** Suryanti Suryanti

**Affiliations:** 1 Universitas Dian Nuswantoro, Semarang, Indonesia

This study explores how knowledge sharing mediates the relationships between transformational leadership, organizational culture, and nursing innovation among registered nurses in the Makkah Health Cluster. The topic is relevant to nursing management and organizational research, particularly in healthcare systems undergoing organizational change.

 The manuscript is generally well structured, and the objectives are clearly presented. The literature provides an appropriate background for the proposed conceptual model, with references that are relevant to the study.

 The cross-sectional design and the use of PLS-SEM are appropriate for the research questions. The sampling procedure, measurement instruments, and analytical approach are described in sufficient detail to understand how the study was conducted. The reporting of the measurement and structural models follows established PLS-SEM practice.

 The results are presented in a clear manner, and the interpretation is consistent with the reported findings. The discussion places the findings in the context of previous studies and acknowledges the main limitations of the study, including the cross-sectional design and the use of self-reported data.

 Ethical approval, reporting guidelines, and data availability are adequately addressed. Based on the information provided, I did not identify major methodological or reporting issues that would affect the overall scientific soundness of the manuscript.

Is the work clearly and accurately presented and does it cite the current literature?

Yes

If applicable, is the statistical analysis and its interpretation appropriate?

Yes

Are all the source data underlying the results available to ensure full reproducibility?

Yes

Is the study design appropriate and is the work technically sound?

Yes

Are the conclusions drawn adequately supported by the results?

Yes

Are sufficient details of methods and analysis provided to allow replication by others?

Yes

Reviewer Expertise:

patient safety, healthcare service, healthcare quality, public health, medical education

I confirm that I have read this submission and believe that I have an appropriate level of expertise to confirm that it is of an acceptable scientific standard.

## References

[1] : Effect of transformational leadership on job satisfaction and patient safety outcomes. *Nursing Outlook* .2018;66(2) : 10.1016/j.outlook.2017.10.004180-189 10.1016/j.outlook.2017.10.004 29174629
